# Transient Cytolysis after Transarterial Chemoembolization in Patients with Hepatocellular Carcinoma

**DOI:** 10.3390/jpm12101663

**Published:** 2022-10-06

**Authors:** Manuel Rodríguez-Perálvarez

**Affiliations:** 1 Department of Hepatology and Liver Transplantation, Hospital Universitario Reina Sofía and IMIBIC, 14004 Córdoba, Spain; h02ropem@uco.es; Tel.: +34-957010328; 2 Centro de Investigación Biomédica en Red de Enfermedades Hepáticas y Digestivas (CIBERehd), 28029 Madrid, Spain; 3 Department of Medicine, Universidad de Córdoba, 14004 Córdoba, Spain

Transarterial chemoembolization (TACE) is a minimally invasive radiological procedure which consists of infusing a chemotherapeutic agent in the main arterial supplier of the liver tumor, usually emulsion-based doxorubicin, followed by the occlusion of the involved vessel with 100–500 micron-sized embolic particles. The direct toxic effect of doxorubicin and the ischemic damage display a dual synergic tumoricidal effect which provokes cell apoptosis and immunogenic changes in the tumor microenvironment, hampering oncogenesis [1]. TACE takes advantage of the hypervascularity of hepatocellular carcinoma (HCC) which, in contrast to the normal liver parenchyma, obtains its blood supply mainly from the hepatic artery and marginally from the portal vein. The true challenge of this technique is to treat the tumor area selectively, minimizing the damage in the surrounding liver parenchyma to decrease the risk of complications and liver decompensation. This supra-selective approach allows the treatment of multiple tumors in patients with chronic liver disease. TACE is considered the standard of care for patients with intermediate-stage hepatocellular carcinoma (HCC) [2,3], which is defined as multinodular disease confined into the liver, in absence of macrovascular invasion and with preserved liver function [4]. In addition, locoregional therapies such as TACE are recommended for patients with early-stage HCC included in the waiting list for liver transplantation unless technically unfeasible [3]. This strategy, usually referred to as bridging, allows the prevention of tumor progression and drop-out. 

The therapeutic effect of TACE is assessed one month after the procedure using a liver dynamic imaging technique, mainly magnetic resonance or computed tomography. Patients are then classified according to the Modified Response Evaluation Criteria in Solid Tumors (mRECIST) [5], which assess changes in tumor size and vascularity compared to baseline, in the following categories: complete response, partial response, stable disease, and progressive disease. TACE is not considered a curative therapy in patients with HCC. Although complete tumor response (i.e., according to mRECIST) is achieved in a significant number of patients, tumor recurrence or development of new lesions is frequent, and median overall survival after TACE ranges between 20 and 36 months [6]. Indeed, overall survival after TACE is modestly prolonged compared to best supportive care (median 20 months vs. 16 months) [7]. However, those patients who are qualified to receive a curative rescue therapy after TACE, mainly surgical resection or liver transplantation, may obtain a much more pronounced survival benefit. 

Predicting radiological response after TACE is of outmost importance as it would allow for individualizing the surveillance strategy. In addition, patients with a priori high risk of incomplete tumor response after TACE could benefit from future strategies including adjuvant systemic agents, which are currently under investigation in randomized controlled trials. There are some clinical characteristics or tumor features associated with poor response to TACE, including number and diameter of the HCC nodules, the Child–Pugh class (standing for liver function), and serum alpha-fetoprotein, either alone or combined in prognostic indexes such as mHAP-II [8] and SNACOR [9]. However, although useful to decide in a certain patient whether new TACE sessions would be appropriate after a first attempt with partial response, these are not accurate enough to guide decisions on the optimal surveillance strategy or regarding eventual adjuvant systemic therapies.

In this Special Issue published in the Journal of Personalized Medicine, A. Granito et al. present the TRANS-TACE study [10], which is an observational analysis of a consecutive cohort of 70 patients with cirrhosis and HCC who received a first session of TACE in a single institution. The authors investigated the influence of dynamic changes in aminotransferases (or transaminases) on the radiological response of the tumor observed one month after the procedure. Transient cytolysis within the first 48 h after TACE, defined as aspartate aminotransferase (AST) increase >46% and/or alanine aminotransferase (ALT) increase >52% from baseline, were associated with increased likelihood of complete radiological response of target lesions at one month (OR = 1.84; 95% confidence interval -CI-1.17–2.98 and OR = 1.42; 95% CI 1.18–2.75, respectively) after controlling for potential confounders. In these series, post-embolization syndrome occurred in 31% of patients. Although the study has inherent limitations such as retrospective design, limited sample size, and lack of external validation, it also shows noticeable strengths including a homogenous cohort from a single center widely experienced in interventional radiology procedures and a multivariable logistic regression analysis to control for baseline tumor features and patient’s characteristics. The study provides eminently practical conclusions for daily clinical practice. First, transient cytolysis is a frequent phenomenon after TACE but elevation of transaminases use was found to be mild or moderate: median AST 150 U/L, median ALT 122 U/L. Second, a 50% increase in transaminases early after TACE carries a probability of complete radiological response of the target lesions as high as 90%. 

Previous studies have suggested a relationship between elevated transaminases and worse oncological outcomes in patients with HCC undergoing TACE (Table 1). However, these studies considered transaminases before TACE, either alone or as part of composite scores such as AST-to-platelet ratio [11,12], AST-to-lymphocyte ratio [13], ASARA [14], or ART scores [15]. In addition, the rates of post-TACE syndrome are usually not reported. The study by Granito et al. [10] brings up a new concept in the setting of supra-selective TACE: mild transient cytolysis after TACE does not provoke a meaningful deterioration of the liver function and could be even beneficial in oncological terms. 

Transient cytolysis after TACE is observed in approximately 30% of patients [18] and may respond to ischemic damage of the peritumoral liver parenchyma. This effect is unintended and indeed TACE trends to be more supra-selective as technical developments allow to identify the arterial tributaries of the tumor in order to avoid complications, including post-TACE syndrome, liver abscesses, or ischemic cholecystitis. However, it is important to note that the periphery of the tumor, including the non-tumoral surrounding parenchyma, shows a more intense expression of proliferative pathways such as the mammalian target of rapamycin [19]. The ischemic effect on the tumor edge would contribute to re-program the immunological microenvironment for more efficient identification and destruction of remnant tumor cells [1,20]. In contrast, if the edge of the tumor is kept uninjured, HCC would be more likely to recur early after TACE and to maintain its spreading capacity [21]. This would explain the relationship between transient cytolysis after TACE and better oncological outcomes. The optimal balance between a supra-selective approach to preserve liver function and an aggressive strategy to maximize anti-tumor effect needs to be established and probably individualized according the functional liver reserve in each patient. For instance, in patients included in the waiting list for liver transplantation in whom TACE is used as a bridging therapy, the oncological benefit should be prioritized over the preservation of liver function, and therefore, a more aggressive TACE approach would be preferred. In contrast, in a patient with intermediate-stage HCC—not a candidate for liver transplantation—with mildly impaired liver function at baseline, a more conservative approach would be required.

Future studies with larger sample size and more prolonged surveillance are required to externally validate the findings by Granito et al. [10] to ascertain to what extent transient cytolysis is beneficial and the optimal threshold of cytolysis beyond which the risk of liver decompensation increases. In any case, monitoring transaminases after TACE while the patient is still admitted in the hospital may be useful to anticipate the biological behavior of HCC. This information would allow the delineation of a personalized TACE approach and, more importantly, enable practitioners to stratify patients according to the risk of adverse oncological outcomes to receive adjuvant systemic therapies whenever they become available.

## Figures and Tables

**Table 1:** List of studies evaluating the role of transaminases on outcomes after transarterial chemoembolization.

First Author, Year	N	UninodularDisease (%)	Child–Pugh A (%)	Post-TACE Syndrome (%)	Role of Transaminases
Granito, 2022 [10]	70	61.5%	84.3%	31.4%	AST and/or ALT increase >50% from baseline were independent predictors of complete radiological response
Jia, 2022 [14] and Nam, 2020 [16]	1336	NR	83.6%	NR	The ASARA score, which includes AST, predicts overall survival after TACE
Tang, 2018 [11]	315	20.3%	98.8%	NR	AST-to-platelet ratio predicted radiological response
Zhu, 2018 [12]	351	86.3%	78.6%	NR	AST-to-platelet ratio and ALT predicted disease-free survival
Liu, 2017 [17]	760	41.2%	81.7%	NR	AST-to-ALT ratio predicts overall survival.
Ha, 2016 [15]	153	25.5%	76.5%	NR	The ART score, which includes AST, predicted overall survival
Yang, 2015 [13]	189	58.7%	87.3%	NR	AST-lymphocyte ratio index independently associated with overall survival

AST: aspartate aminotransferase; ALT; alanine aminotransferase; NR: not reported; TACE: Transarterial chemoembolization.

## Data Availability

Not applicable.
